# Evaluation of Female Patients Motivating Factors for Aesthetic Surgery 

**Published:** 2012-07

**Authors:** Seyed Mehdi Moosavizadeh, Feizollah Niazi, Abdoljalil Kalantar-Hormozi

**Affiliations:** 1 Department of Plastic Surgery, 15th Khordad Hospital, Shahid Beheshti University of Medical Sciences, Tehran, Iran;; 2 Department of Plastic Surgery, Modarres Hospital, Shahid Beheshti University of Medical Sciences, Tehran, Iran

**Keywords:** Aesthetic surgery, Female, Motivational Factors, Iran

## Abstract

**BACKGROUND:**

Nowadays, plastic surgeries are among the most popular types of surgeries around the world and Iran has one of the highest rankings in this respect regarding its population size. Based on these facts, the present study was conducted in order to evaluate the volunteers’ motivations attitudes and informational sources in Tehran, Iran.

**METHODS:**

The study was conducted on a cross-sectional basis. All patients who underwent plastic surgeries during years 2005 and 2006 were evaluated and all the required information was recorded and statistically analyzed.

**RESULTS:**

Seventy five patients were enrolled. Their average age was 33±13 years (minimum of 17 and maximum of 63 years old). Rhinoplasty (52%) and abdominoplasty (8%) were the most and least common performed surgeries, respectively. The major important motivators were family, friends, classmates, and colleagues (40%) and the least were magazines and journals (4%).10.7% oftheparticipants described theposture and function of the target organs as perfect, but they planned to improve its aesthetic or functional aspects by surgery.

**CONCLUSION:**

Those who achieve their information of aesthetic surgery from mass media have a better understanding of this field. Also, the motivational stimuli for performing aesthetic surgeries have shown to be different from what were previously deemed.

## INTRODUCTION

Aesthetic surgeries are the most common types of surgeries around the world which have increasing trend. In the United States, 1.3 million people underwent aesthetic surgeries in 2000, which indicates an almost two folds increase since 1992.^1^Althoughitisonly6decadessinceaestheticsurgerywas performed in Iran, this field of surgery is now one of the most dynamic fields of surgery and medical research in Iran and has a promising future.^2^Since many of the aesthetic surgeries are conducted in private clinics and doctors’ offices, there are no exact statistics of the number of performed aesthetic surgeries in this country. Stillbased ontheexisting records, Iran has one of the highest rankings in this respect regarding its population size.^3^

Despitethepopularityandprevalence of this type of surgery, no actual study has been conducted onthemotivations andpointsof view of people for doing such surgeries in Iran. Most of reasons and motivations behind doing aesthetic surgeries in various societies are different.^4^Although most patients achieve their information about aesthetic surgeries from media, journals, friends and family,^4^the ratio of each has not been estimated for this country. Consequently, thefollowing studywasconducted in order to find out the motivations and informational resources offemale volunteers of aesthetic surgery in a referral center in Tehran, Iran during the years 2005 and 2006.

## MATERIALS AND METHODS

In a cross-sectional study, all volunteers patients were surveyed. In this sense, all female patients who had referred to our center affiliated to Shahid Beheshti University of Medical Sciences, Tehran, Iran during the years 2005 and 2006 for aesthetic surgery were interviewed and underwent clinical examination. Written consent was obtained from all patients prior to their inclusion in the study, and all data regarding patient’s characteristics, motivations for aesthetic surgery, duration from their decision-making until referral, the resources of their motivation or stimuli and their point of view of aesthetics of the target organ were recorded.

All patients who declined to cooperate after the initial consent were excluded from the study. Afterwards, the findings were statistically analyzed and the results were presented by descriptive and analytical statistics.

## RESULTS

**Fig. 1:**
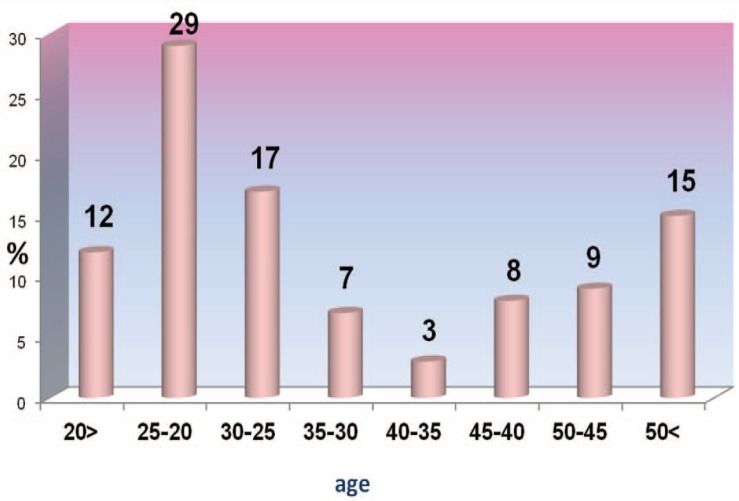
Frequency of distribution based on age for aesthetic surgery

Two hundreds and two female patients under- went aesthetic surgery during the years 2005 and 2006 in our center, from which 106 patients (52%) gave an initial consent to participate in this study. However, only 75 (37%) patients cooperated until the end of the study after their surgery and the rest were excluded. The patients’ average age was 33±13 years (Range:17-63yearsold).Themedianand mode of the population were 28 and 21 respectively. In addition, percentile 25 was equal to 22 years old and percentiles 75 and 90 were equal to 45 and 54 years old respectively. Figure 1 demonstrates the frequency of distribution of the patients based on their age.

Forty eight percent of the participants were single and 52% married (divorced and separated participants were included in the singles group). The duration from when the participants had become interested in aesthetic surgery until the time of their referral was 3.2±3.3 years. The least duration of decision-making was one year and longest was 22 years. Percentiles 25, 50, 75 and 90 were 1, 2, 4, and 7 respectively.

Seventy two percent of the participants knew at least one person who had underwent an aesthetic surgery from which, 36% were close family members, 18.7% were distant family members, 17.3% were friends and acquaintances. In addition, 30.7% of the patients knew someone who had underwent aesthetic surgery specifically in our center, from which 21.3% had stated that they had an excellent result and the rest had no exact information of their family member or acquaintances opinion of his/her surgery.

**Fig. 2:**
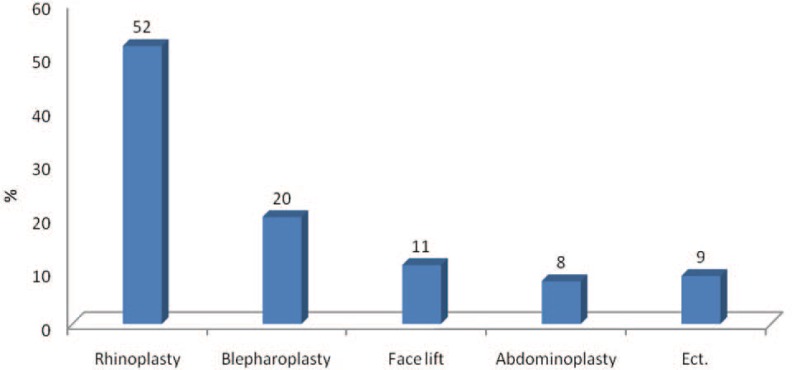
Frequency of distribution based on type of surgery.

In this study, 9.3% of the conducted surgeries were done due to medical reasons, 60% due to aesthetics and cosmetic reasons and 30.7% because of both. 45.3% of the patients had college or high school degree, the same percent had undergraduate and graduate degree, and 9.4% had less than high school or no degree. 37.3% of the studied population were housewives orunemployed, 24%werestudents, 30.7% were employees in public service and about 8% were freelance workers (Figure 2).

**Fig. 3:**
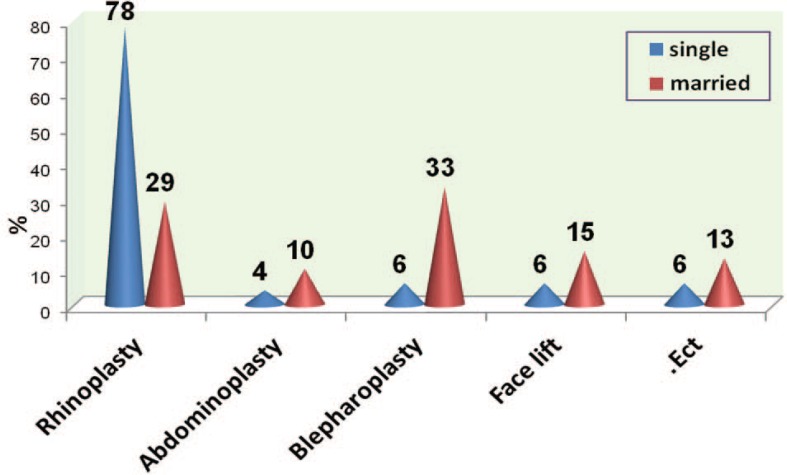
Frequency of distribution based on marring status

**Fig. 4:**
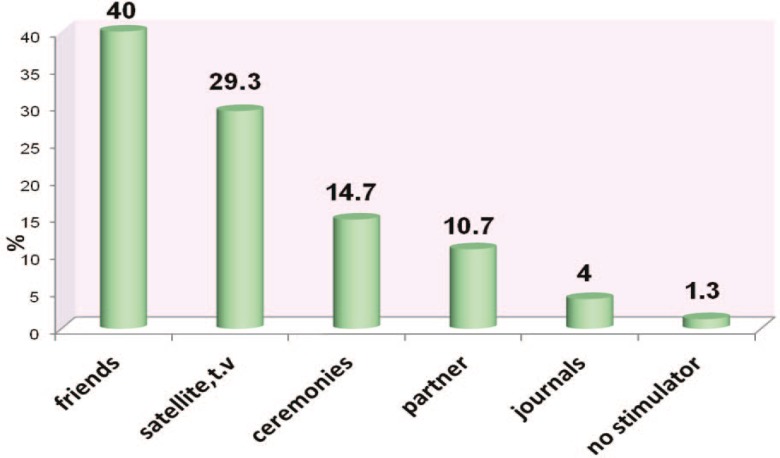
Frequency of distribution based on motivating factors.

Figure 3 demonstrates the frequency of different kinds of a esthetic surgeries based on marital status. In this sense, the most common aesthetic surgery in single and married participants was rhinoplasty (77.8%) and blepharoplasty (33.2%), respectively. The main motivating factors for aesthetic surgeries were family, friends, classmates, and colleagues (40%) and the least motivating factors were journals and magazines (4%) (Figure 4).

In analyzing the motivating factors based on age, the main stimuli or motivations for doing aesthetic surgeries have been family, friends, classmates, and colleagues(66.7%) and television, satellite, and cinema (33.3%) in participants under 20 years of age. In age range of 20-25 years old, the motivating factors were family, friends, classmates, and colleagues (50%) plus television, satellite, and cinema (31.8%). The rest of the motivating factors, such as journals and magazines, had a little share. In age range of 25-30 years old, the motivating factors were family, friends, classmates, and colleagues (30.8%), patient’s partner (30.8%), and television, satellite, and cinema (23.1%). In age range of 30-35 years old, parties, ceremonies and anniversaries (40%) were the most effective motivational factors. Patient’s partner (20%), television, satellite, and cinema (20%), and journals and magazines (20%) were next in the line. In age range of 35-40 years old, the motivational factors were family, friends, classmates, and colleagues (50%) and television, satellite, and cinema (50%).

In age range of 40-45 years old, the motivating factors were family, friends, classmates, and colleagues (50%), patient’s partner (16.7%), television, satellite, and cinema (16.7%) and parties, ceremonies and anniversaries (16.7%). In age range of 45-50 years old, the motivating factors were television, satellite, and cinema (42.9%) and parties, ceremonies and anniversaries (42.9%), and patient’s partner (14.3%). In ages above 50 years old, the motivating factors were family, friends, classmates, and colleagues (45.4%), television, satellite, and cinema (27.3%) and parties, ceremonies and anniversaries (27.3%). Table 1 and 2 has details on the motivational factors of female volunteers for aesthetic surgery based on their education and career.

**Table 1:** Motivation source for plastic surgery based on patients occupation

**Job**	**Motivation source**	**No.**	**%**
Clerk	Without any motivation	1	4.3
	Friends and classmate	10	43.6
	TV, sat, movie actors, artist	6	26.1
	Magazines and journals	1	4.3
	Party	4	17.4
	Partner, spouse	1	4.3
	Total	23	100
Free	Friendsand classmate	3	50
	Magazines and journals	1	16.7
	Partner, spouse	2	33.3
	Total	6	100
Housekeepers	Friends and classmate	6	21.4
	TV, sat, movie actors, artist	9	32.1
	Magazines and journals	1	3.6
	Party	7	25
	Partner, spouse	5	17.9
	Total	28	100
Student	Friends and classmate	11	61.1
	TV, sat, movie actors, artist	7	38.9
	Total	18	100

**Table 2:** Motivation source for plastic surgery based on patients education level

**Level of education**	**Motivation source**	**No.**	**%**
Mid-school	Friends and classmate	2	28.6
	TV, sat, movie actors, artist	1	14.3
	Magazines and journals	1	14.3
	Party	2	28.6
	Partner, spouse	1	14.3
	Total	7	100
High-school	Friends and classmate	14	41.2
	TV, sat, movie actors, artist	10	29.4
	Party	7	20.6
	Partner, spouse	3	8.8
	Total	34	100
BSC or higher	Friends and classmate	14	41.2
	TV, sat, movie actors, artist	11	32.4
	Magazines and journals	2	5.9
	Party	2	5.9
	Partner, spouse	4	11.8
	Without any motivation	1	2.8
	Total	34	100

In married participants, the motivational factors for performing aesthetic surgeries were television, satellite, and cinema (30.8%), family, friends, classmates, and colleagues (28.2%), parties, ceremonies and anniversaries (20.5%), and patient’s partner (17.9%) totally. In single participants, the motivational factors for performing aesthetic surgeries were family, friends, classmates, and colleagues (52.8%), television, satellite, and cinema (27.8%), parties, ceremonies and anniversaries (8.3%), journals and magazines (5.6%), and patient’s partner (2.8%) on the whole. 2.8% of the participants reported no special motivational factor for doing aesthetic surgery. The most important motivational factors for patients who underwent rhinoplasty had been family, friends, classmates, and colleagues (43.6%). The most important motivational factors for patients who underwent abdominoplasty had been patient’s partner (33.3%) and family, friends, classmates, and colleagues (33.3%).

The most significant motivational factors for patients who had undergone meloplasty were family, friends, classmates, and colleagues (50%). On the other hand, the most significant motivational factors for performing blepharoplasty were parties, ceremonies and anniversaries (33.3%) and television, satellite, and cinema (33.3%). Also, parties, ceremonies and anniversaries (42.9%) and family, friends, classmates, and colleagues (42.9%) were the significant motivational factors for performing other kinds of aesthetic surgeries. About 10.7% of the patients who underwent aesthetic surgery in this study considered themselves very attractive, 41.3% attractive, 41.3% somewhat attractive, and 6.7% not attractive. No one chose the ugly item. 45.3% of the participants believed that the target organ was not much bad in appearance and function, however it should become better. 32% believed that the target organ had a bad appearance and 12% had actual problems with the function or appearance of the target organ in their daily lives. 10.7% of the participants regarded the target organ as good in appearance and function, but wanted to make it excellent or become similar to a celebrity.

**Fig. 5:**
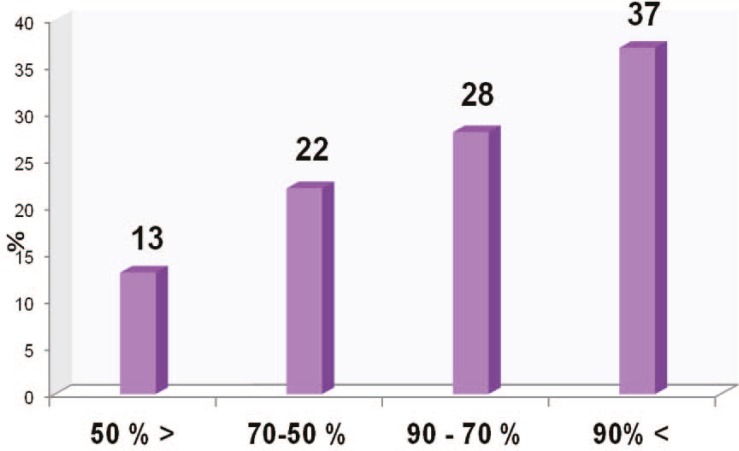
Patients anticipation of aesthetic result and success.

Figure 5 shows the patients’ anticipation of their own aesthetic surgery result and how successful it can be. 66.6% of those who underwent rhinoplasty anticipated that their surgery would be successful morethan70%. Those who underwent abdominoplasty, blepharoplasty, lifting and other kinds of aesthetic surgeries anticipated that their operations would be successful more than 50%, 80%, 62.5% and 42.9%, respectively. 74.4% of married participants anticipated that their operation would be successful more than 70%, while this percentage was 55.6% in single participants.

## DISCUSSION

Inthisstudy,41%oftheparticipants were teenagers and young people (under 25 years old),and65%wereunder35yearsold. The sestatistics are different from the results of western studies in which only 27% of the volunteers for aesthetic surgery have been under 34 years old^4^or 64% of the volunteers have been between 31 to 50 years old.^5^In other words, the demographic distribution of people volunteering for a esthetic surgeries in Iran is much younger than some western countries.

About 30.7% of the patients knew at least one person (a friend, family member, classmate or colleague) who had done aesthetic surgery, but 69.3% of them had no idea about the final result of that person’s operation. In other words, they had decided to undergo aesthetic surgery without first finding out about the final result of their friend, family member, classmate or colleague’s surgery. This attitude could be the result of that^1^enough time has not passed for the final result to show up^3^ and performing aesthetic surgery itself can motivate others to do it themselves, without considering its results on the first person. It has become obvious that publicity and acquaintances’ recommendations are more effective than logic explanations and research for motivating one to perform aesthetic surgery. Consequently, many people in Iran approach to aesthetic surgery without knowing other people’s final results. 

Rhinoplasty (52%) has been the most common kind of performed aesthetic surgery and abdominoplasty (8%) the least common. However, liposuction, breast surgeries, rhinoplasty and blepharoplasty had been the most common aesthetic surgeries in other studies.^6^,^7^ In the studies of other countries, 96% of the patients had named journals, magazines and television as their motivation for performing aesthetic surgery.^3^Such results are evidences of cultural differences between Iran and other countries and show that journals and magazines play a less important role in transferring information and persuasion.

In the present study, the patients’ motivations were not dependant on their education or kind of surgery. However, they were dependant on the volunteers’ career or marital status. Still, similar to another study, patients who had achieved their information of aesthetic surgery from mass media (e.g. student sand housewives) had a better understanding of this field.^4^The stimulus for performing aesthetic surgery was also dependant on the target organ and patients point of view about that organ.

Since those patients who achieved their information from the mass media had a better understanding of aesthetic surgeries, it is recommended that a thorough and united approach be taken to transfer the right and sufficient information to those who feel the need to know about aesthetic surgeries.

The results of this study might not be generalized since the studied population was only the female patients who had been volunteered to undergo aesthetic surgery in our center in Iran. It is suggested that similar study to be undertaken with a larger population, possibly in the whole country, in order to derive out people’s actual point of view and motivating factors regarding aesthetic surgery.
